# Neuromyelitis Optica Spectrum Disorders Resembling Wernicke’s Encephalopathy: A Case Report and Review of Literature

**DOI:** 10.7759/cureus.63920

**Published:** 2024-07-05

**Authors:** Takuya Saito, Ken Nakano, Tsuyoshi Uchiyama

**Affiliations:** 1 Department of Neurology, Seirei Hamamatsu General Hospital, Hamamatsu, JPN

**Keywords:** immunotherapy, radiology, encephalopathy, brain diseases, wernicke encephalopathy, neuromyelitis optica

## Abstract

Both neuromyelitis optica spectrum disorder (NMOSD) and Wernicke's encephalopathy (WE) involve brain lesions. However, their treatments are quite different. In this report, we describe the case of a 29-year-old woman with NMOSD, who presented with clinical and imaging findings similar to those of WE. She was admitted to our hospital with a headache, vomiting, and loss of appetite for two weeks and diplopia for nine days. Magnetic resonance imaging revealed lesions in the area postrema, periaqueductal gray matter, thalamus, and right frontal lobe. Vitamin B1 supplementation was ineffective. The patient was diagnosed with NMOSD because serum aquaporin-4 antibody was detected after admission. Her symptoms improved with immunotherapy. The possibility of NMOSD should be considered in patients with suspected WE.

## Introduction

Neuromyelitis optica spectrum disorder (NMOSD) and Wernicke's encephalopathy (WE) are two distinct neurological conditions that can present with overlapping clinical and radiological features. NMOSD is an autoimmune disorder characterized by inflammation and demyelination of the central nervous system. Severe optic neuritis and myelitis attacks are common, and brain lesions can occur [1,2]. The response of astrocytes to aquaporin-4 (AQP4) antibodies causes inflammation and severe neuronal injury [3]. Therefore, AQP4 antibody testing is a useful diagnostic tool [4]. Early diagnosis is important because immunotherapy can improve symptoms in the acute phase [5] and prevent recurrence in the chronic phase [2].

In contrast, WE is a neurological disease caused by vitamin B1 deficiency, commonly associated with chronic alcoholism, malnutrition, and other conditions impairing nutrient absorption. WE primarily affects the periaqueductal gray matter, midbrain, hypothalamus, and thalamus leading to characteristic symptoms such as ocular abnormalities, ataxia, and global confusion [6,7]. Serum vitamin B1 levels are useful for the diagnosis of WE; however, it takes days and lacks sensitivity and specificity. Hence, if WE is suspected based on clinical symptoms, immediate vitamin B1 supplementation is essential [8]. Despite their distinct etiologies, both NMOSD and WE can present with brain lesions visible on magnetic resonance imaging (MRI), making differential diagnosis challenging. Accurate and timely differentiation is critical, as the treatments for these conditions differ significantly.

Here, we report a case of NMOSD with clinical and imaging findings similar to those of WE. In addition, we reviewed previous case reports of NMOSD resembling WE.

## Case presentation

A 29-year-old Japanese woman without any significant medical history developed a headache two weeks prior to presentation, followed by nausea and loss of appetite. She had developed diplopia nine days previously and had visited a hospital five days previously. WE was suspected based on the patient’s history and MRI findings, and vitamin B1 supplementation was initiated. The patient was referred to our hospital because her symptoms did not improve.

**Figure 1:**
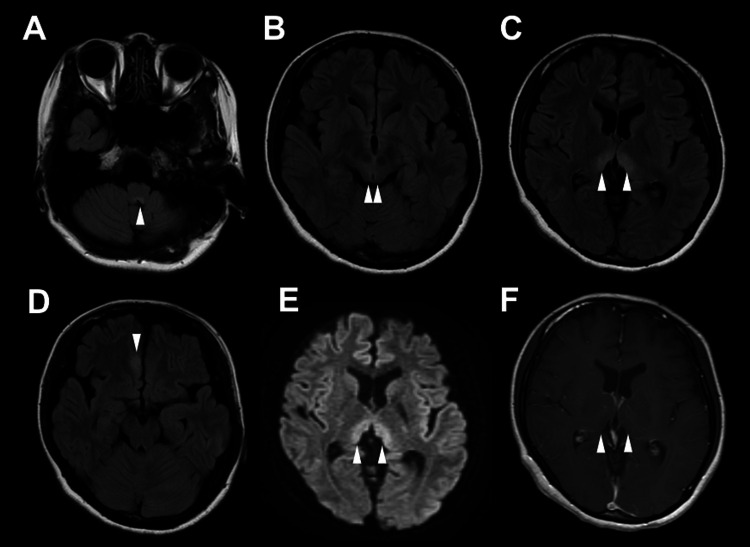
Magnetic resonance imaging findings Fluid-attenuated inversion recovery images show high-signal lesions in the bilateral area postrema (arrowhead in A), bilateral periaqueductal gray matter (arrowheads in B), bilateral medial thalamus (arrowheads in C), and medial part of the right frontal lobe (arrowhead in D). Diffusion-weighted imaging shows high-signal lesions in the bilateral medial thalamus (arrowheads in E), although no contrast enhancement (arrowhead in F).

The patient presented with somnolence, right oculomotor nerve palsy, deep tendon reflex hyperreflexia, and stuttering. Pregnancy test results were negative. Blood glucose, vitamin B1, vitamin B12, and folic acid levels were normal, as were thyroid and adrenal functions. Screening tests for infectious diseases, including tests for herpes simplex virus, varicella-zoster virus, hepatitis B virus, hepatitis C virus, and human immunodeficiency virus, were negative. Antinuclear antibodies, double-stranded deoxyribonucleic acid, ganglioside GM1, ganglioside Q1b, Sjögren syndrome antigen A, Sjögren syndrome antigen B, soluble interleukin-2 receptor, glutamic acid decarboxylase, thyroid peroxidase, and thyroglobulin antibodies were negative. Cerebrospinal fluid (CSF) examination revealed pleocytosis (26 cells/μL; 25 monocytes, 1 polymorphonuclear neutrophil) and slightly elevated protein levels (43 mg/dL). Oligoclonal band was negative. Immunoglobulin G (IgG) index was 1.1. MRI showed high-signal lesions in the bilateral area postrema, bilateral periaqueductal gray matter, bilateral medial thalamus, and medial part of the right frontal lobe on fluid-attenuated inversion recovery images (Figure 1). MRI showed no spinal cord or optic nerve lesions.

WE was suspected based on episodes of poor nutritional intake and symptoms of ocular abnormalities and global confusion. She was initially treated with vitamin B1, although her symptoms did not improve. We suspected autoimmune encephalitis due to subacute progressive neurological symptoms and CSF pleocytosis [9], and started administration of high-dose intravenous methylprednisolone (1 g for five days). Serum AQP4 antibody positivity in an enzyme-linked immunosorbent assay was found on day 7 after admission, and the patient was diagnosed with encephalitis due to NMOSD. Plasma exchange and immunoglobulin therapy were administered, and her symptoms improved.

## Discussion

We reported the case of a patient with NMOSD whose clinical and radiological findings were similar to those of WE. Pleocytosis and AQP4 antibody positivity led to the diagnosis of NMOSD. The patient’s symptoms improved after the immunotherapy.

NMOSD is caused by the downregulation of glutamate transporters via the formation of AQP4-IgG complexes, resulting in glutamate accumulation, cellular edema, and astrocyte injury. AQP4-IgG complexes bind to the foot process of astrocytes, resulting in demyelination and axonal injury [3]. Contrastingly, WE is caused by brain injury due to impaired citric acid cycle due to vitamin B1 deficiency [8]. Furthermore, Yang et al. reported vitamin B1 deficiency induces astrocytotoxicity by regulating glutamate transporter expression and inducing inflammatory cytokines [10]. NMOSD and WE might be similar in that they result in astrocyte injury and consequent blood-brain barrier failure [10]. This similarity in causing astrocyte injury might explain why NMOSD can present with clinical and radiological findings that mimic WE. NMOSD with only brain lesions has been reported to resemble WE [11].

Cases requiring differentiation between NMOSD and WE have been reported (Table 1) [10-17]. The distinction between NMOSD and WE could be learned from these cases. First, regarding the neurological findings, the three major signs of WE, ocular abnormalities, ataxia, and gross confusional state, are often present in NMOSD and may not be useful for differentiation. Second, regarding the imaging findings, optic neuritis and spinal cord lesions are less likely to occur with WE and may be useful for differentiation. Third, regarding the laboratory findings, measurement of vitamin B1 and AQP4 antibodies may be useful although it takes time to obtain test results, and false positives for AQP4 antibodies have been reported in 1.3% of healthy subjects [18]. As in this case, pleocytosis could occur in NMOSD. In the two patients diagnosed with WE, a 28-year-old woman was AQP4 antibody-positive six months after discharge [16], and a 20-year-old woman was AQP4 antibody-negative after steroid administration [17]. In both cases, NMOSD could have been diagnosed had AQP4 antibody been tested upon admission. Although most reported cases are of women in their 20s, NMOSD can occur in men and in older age [19]. Some patients diagnosed with WE, having older onset, who do not respond to treatment were reported [20]. Some of these patients might actually be patients with NMOSD. In the future, more rapid testing and differentiation methods will be needed for NMOSD and WE.

**Table 1:** Reported cases requiring differentiation between neuromyelitis optica spectrum disorder and Wernicke's encephalopathy A, ataxia; AQP4, aquaporin-4; CSF, cerebrospinal fluid; Dx, diagnosis; F, female; GC, global confusion; M, male; NMOSD, neuromyelitis optica spectrum disorder; OA; ocular abnormalities; ON, optic neuritis; UL, unilateral limb symptom; UNK, unknown; WE, Wernicke's encephalopathy; +, positive; -, negative

Dx	Age Sex	Time course	Anorexia or nausea	Symptoms like WE	Symptoms like NMO	Brain lesions like WE	Spinal lesions	CSF	Vitamin B1 deficiency	AQP4 antibody	Reference Number
NMOSD	21F	Acute	+	GC	-	+	UNK	Pleocytosis	UNK	+	[10]
NMOSD	48M	UNK	-	GC	-	+	-	Normal	UNK	+	[11]
NMOSD	26M	UNK	+	GC	-	+	-	Normal	UNK	+	[11]
NMOSD	23F	Acute	+	-	ON	+	UNK	UNK	-	+	[12]
NMOSD	28F	Subacute	+	OA, A, GC	UL	+	-	Pleocytosis	UNK	+	[13]
NMOSD	20F	Acute	+	OA, A, GC	ON, UL	+	+	Normal	UNK	+	[14]
NMOSD and WE	17F	Subacute	+	OA, A	ON	+	UNK	Elevated protein level	UNK	+	[15]
WE	24F	Acute	+	GC	-	+	UNK	Normal	+	-	[10]
WE	28F	Subacute	+	OA, A	-	+	UNK	UNK	UNK	+	[16]
WE	20F	Subacute	+	OA, A, GC	ON	+	UNK	Normal	UNK	-	[17]

## Conclusions

We reported the case of a patient with NMOSD who presented with symptoms and MRI findings similar to WE. Her symptoms did not improve with vitamin B1 administration and improved with immunotherapy. Clinical signs and imaging findings of NMOSD and WE might be similar, and close examination is necessary for differentiation. The possibility of NMOSD should be considered in patients with suspected WE since WE and NMOSD have different therapeutic modalities.
